# Healing Mechanisms in Cutaneous Wounds: Tipping the Balance

**DOI:** 10.1089/ten.teb.2021.0114

**Published:** 2022-10-11

**Authors:** Adam J. Singer

**Affiliations:** Department of Emergency Medicine, Renaissance School of Medicine, Stony Brook University, Stony Brook, New York, USA.

**Keywords:** acute wound healing, inflammatory response, regenerative medicine, regenerative healing, reparative healing, scarring, stem cells

## Abstract

Acute and chronic cutaneous wounds pose a significant health and economic burden. Cutaneous wound healing is a complex process that occurs in four distinct, yet overlapping, highly coordinated stages: hemostasis, inflammation, proliferation, and remodeling. Postnatal wound healing is reparative, which can lead to the formation of scar tissue. Regenerative wound healing occurs during fetal development and in restricted postnatal tissues. This process can restore the wound to an uninjured state by producing new skin cells from stem cell reservoirs, resulting in healing with minimal or no scarring. Focusing on the pathophysiology of acute burn wounds, this review highlights reparative and regenerative healing mechanisms (including the role of cells, signaling molecules, and the extracellular matrix) and discusses how components of regenerative healing are being used to drive the development of novel approaches and therapeutics aimed at improving clinical outcomes. Important components of regenerative healing, such as stem cells, growth factors, and decellularized dermal matrices, are all being evaluated to recapitulate more closely the natural regenerative healing process.

**Impact Statement:**

Acute wounds from thermal injury are common; they exert substantial physical and psychological effects on a patient and result in significant morbidity and mortality. This review provides a detailed overview of the mechanisms of reparative and regenerative wound healing; discusses the key cell types, signaling molecules, and molecular targets that influence these important biological pathways; and highlights current therapeutic approaches aimed at promoting regenerative wound healing. An increased understanding of the underlying mechanisms of reparative and regenerative healing will contribute to the development of innovative strategies for the clinical treatment of patients with severe burns.

## Introduction

Cutaneous wounds pose a significant health and economic burden. In 2014, 8.2 million Medicare beneficiaries in the United States filed claims for wound care, and total wound costs were estimated at $28.1 billion to $96.8 billion per year.^1^ The United Kingdom's National Health Service (NHS) spent an average of £2,151 per patient (catchment population of 250,000 adults) with acute wounds in 2012 and 2013.^2^ Although estimates for the global prevalence of chronic wounds vary, studies in Western Europe report that 1% to 2% of the population experience chronic wounds, and the NHS spent £2,870 per patient on chronic wounds in United Kingdom in 2012 and 2013.^2–5^

The primary difference between acute and chronic wounds is the length of time required for wound closure. Acute wounds, including those caused by trauma, iatrogenic wounds (surgical procedures), or accidental wounds (e.g., burns, lacerations, and abrasions), progress in an orderly manner through the healing process.^6,7^ Chronic wounds, including skin ulcers of various etiologies (e.g., venous or arterial disease, pressure, and vasculitis or autoimmune disease), can be prolonged at many points of the healing process by underlying factors (e.g., infection, cell senescence, inflammation, and ischemia) that interfere with healing.^6,8,9^ Most acute wounds heal with minimal sequelae, but even wounds that completely close can lead to adverse consequences for the patient (e.g., excessive scarring can have long-term negative effects and necessitate additional treatment).^6,10,11^

Acute wounds resulting from thermal injury are common and can result in significant morbidity and mortality.^12–14^ An estimated 9 million injuries and 121,000 deaths from fire, heat, and hot substances were reported in the 2017 Global Burden of Disease study.^13^ Morbidity associated with a severe burn injury can persist for 10–20 years after the initial injury.^14,15^ In 2019, average hospital charges for a U.S. patient with a burn injury were estimated at $105,000, which increased to $310,000 for nonsurvivors.^12^

Scarring from large burn wounds or burn wounds in functionally or cosmetically important areas (e.g., the hands, face, neck, and joints) may impart a significant burden on patients.^16,17^ Scars can contribute to contracture in functionally or cosmetically important areas, reduced mobility, pain, itching, disfigurement, reduced quality of life, and psychological consequences (e.g., depression and anxiety).^16,18–21^ Severe burns can also cause extensive nerve damage and peripheral neuropathy.^22^ In a systematic review, patients with burns reported the lowest scores in domains of work and heat sensitivity, bodily pain, physical role limitations, and pain/discomfort over the short term.^23^ Burn injuries are also associated with a high prevalence of posttraumatic stress disorder, ranging from 11% to 50% across studies.^24^

Burn wounds heal through the reparative wound healing process and result in the formation of scar tissue, which is mainly composed of dense collagen, and lacks sweat glands, hair follicles, and other appendages.^10,25^ In contrast, regenerative healing is a process that can restore the wounded skin to an uninjured state, with minimal or no scarring.^26^ This type of healing occurs in early-development fetal wounds (up to 24 weeks of gestation in humans) and results in skin that has a structure and function similar to the surrounding uninjured skin, including regenerated epidermal appendages.^26,27^ While the goal of true regenerative healing has not yet been realized with current therapies, it is the aspirational objective of burn wound treatment.^17,28,29^

This review provides an overview of the mechanisms of reparative and regenerative wound healing; discusses the key cell types, signaling molecules, and molecular targets that influence these pathways; and highlights current and future therapeutic approaches aimed at promoting regenerative healing in acute burn wounds. Recent reviews by Zhao *et al*. and Han and Ceilley provide a robust overview of the current landscape for chronic wounds.^9,11^

## The Wound Healing Process

Most postnatal wounds heal through reparative healing, which is a complex biological process involving cells, signaling molecules, and the extracellular matrix (ECM) that occurs in four overlapping, highly coordinated stages: hemostasis, inflammatory, proliferation, and remodeling.^25,29^

Fetal wounds heal *in utero* through regenerative healing; postnatal microenvironments with an attenuated inflammatory response (e.g., the oral mucosa) also show healing with regenerative characteristics, including a reduced immune response and scarring.^30^ Regenerative healing occurs in the same four stages as reparative healing, with some key differences. The following sections summarize the wound healing process and the important biological components that differ between the two wound healing mechanisms; Table 1 provides more detail on the specific cells and signaling molecules involved in each type of wound repair.

**Table 1.** Key Therapeutic Targets Involved in Reparative Wound Healing and Known Differences in Regenerative Healing

	Reparative healing	Regenerative healing
Hemostasis phase		
Time point^a^	Starting immediately after wounding and lasting a few hours to 2 days^27,200^	Does not occur in regenerative healing; reepithelialization starts immediately after wounding (murine and rat models)^33,40^
Cells	Platelets are activated and drive clot formation, which prevents excessive blood loss and protects the wound from infection^25,35–37^	Not well characterized in the published literature
Signaling molecules	VEGF is released by platelets^201^	Not well characterized in the published literature
ECM	Cross-linked fibrin and fibronectin contribute to clot formation and provide an initial structure for cell movement^25,35,38^	A fibronectin clot forms^39^ Tenascin is present in the tissue surrounding the wound and helps with rapid reepithelialization^33,62^ There are high levels of hyaluronic acid^30^
Inflammatory phase		
Time point	Starts on day 1 during hemostasis and can last up to day 8^27,200^	Although it is known that this phase is attenuated in fetal wounds, the timing of the appearance of cells and cytokines associated with inflammation has not been characterized in human or other large mammalian fetuses^61,62^
Cells	Toll-like receptors on damaged cells trigger the innate immune response^43,44^ Leukocytes protect the wound from infection^46^ Neutrophils secrete signaling molecules to debride the wound, degrade the clot, attract additional inflammatory cells, and contribute to angiogenesis^46,49,54,55,133,202–204^ M1 macrophages clear debris from the wound^71,133,205^ Mast cells reduce blood coagulation and increase fluid accumulation^52,53^ Natural killer cells and plasmacytoid dendritic cells contribute to antimicrobial activity, angiogenesis, and tissue repair^56–59^	Few inflammatory cells are present; larger or more severe wounds may elicit a stronger inflammatory response^40,41,62,63^ Macrophages are present, but are not responsive to the wound^63^ Mast cells may be present, but are not activated^64^
Signaling molecules	Inflammatory cytokines and chemokines (e.g., TNF-α, TGF-β1, IL-1, IL-6, and IL-8) promote the migration of immune cells to the site of inflammation^49,51,133,203,206,207^ Proteases debride the wound and eliminate toxins from damaged tissue^46,47^ Growth factors (e.g., HGF, VEGF, and FGF) promote angiogenesis^49,54,55^ Histamine and heparin reduce blood coagulation and increase fluid accumulation^52,53^ Type I interferons contribute to wound healing and antimicrobial activity^56,57^	Expression of inflammatory cytokines and chemokines, including IL-6 and IL-8, is reduced or absent^65–67^ Anti-inflammatory IL-10 expression is increased^68,69^
ECM	New blood vessels start to form^49,54,55^	Angiogenesis does not increase, and does not contribute to, inflammation^40,62^
Proliferation phase		
Time point	Starts 3–10 days after wounding and can last until day 25^27,200^	Reepithelialization starts immediately and wound closure is achieved 2–3 days after wounding (murine, rat, and lamb models)^33,40,119^
Cells	M2 macrophages secrete signaling molecules to attract fibroblasts and keratinocytes to the wound^49,71,205^ Fibroblasts migrate and proliferate to deposit the ECM for granulation tissue^72,74,76^ Stem cells or mesenchymal progenitor cells from hair follicles, injured nerves, and the bone marrow, and dedifferentiated cells from underlying fat contribute to tissue generation^79–82^ Endothelial cells and endothelial progenitor cells form new blood vessels^77,78^ Activated mast cells contribute to angiogenesis^208,209^ Fibroblasts differentiate into myofibroblasts rich in α-SMA fibers, which contract to narrow the wound opening and increase vascularization^74,83–86^ Keratinocytes from the surrounding tissue and stem cells from the interfollicular epidermis and hair follicles reepithelialize the wound^81,87–91^	Endothelial progenitor cells originate from the bone marrow and contribute to angiogenesis and increased blood circulation^99^ Increased migration of fibroblasts increases hyaluronic acid content of the ECM^95,98^ Fibroblasts and keratinocytes produce an organized ECM^33,95^ Fibroblasts are resistant to TGF-β1–induced differentiation into α-SMA–positive myofibroblasts^210,211^ Fibroblasts contract and contribute to wound closure^104,107,108^
Signaling molecules	Cytokines (including IL-1 and IL-6), chemokines, and growth factors (including VEGFs and TGF-β) attract fibroblasts and keratinocytes to the wound^49,71^ PlGF helps stimulate angiogenesis of the granulation tissue^212^ TGF-β1 induces fibroblasts to differentiate into myofibroblasts^83,84^	High levels of IL-10: upregulate hyaluronic acid^96,97^; increase migration and invasion of fibroblasts^95,98^; and help regulate the formation of ECM and fibroblast differentiation^66,95,211^ Low levels of VEGF decrease angiogenesis^102^
ECM	Collagen types I and III, fibronectin, hyaluronic acid, and proteoglycans form the ECM of the granulation tissue^72,74,76^	An organized ECM of fibronectin, tenascin, chondroitin sulfate, and hyaluronic acid is produced by fibroblasts and keratinocytes^33,39,62,95^ An actin cable surrounding the wound brings the wound edges closer together^107,109^ Angiogenesis and blood circulation increase, although not to the same degree as in reparative healing^62,99–102^
Remodeling phase		
Time point	Starts around days 21–23 and can last for up to 2 years^27,200,213^	Starts 3 days after wounding and is complete by 14 days (murine model with human skin transplant, lamb model)^119,214,215^
Cells	Fibroblasts, keratinocytes, and inflammatory cells secrete MMPs^111–113^	Fibroblasts lay down collagen in a pattern similar to the surrounding skin^114,118,119,216^
Signaling molecules	MMPs break down the granulation tissue and remodel the ECM into a more permanent structure^111–113^	Higher levels of antifibrotic TGF-β3 than profibrotic TGF-β1 and TGF-β2^30,103,105,106^ IL-10 downregulates the expression of collagen type I^105,106,116,117^
ECM	Collagen is laid down in parallel bundles to form the more permanent ECM and scar tissue^30,114,115^ The ratio of fibrillar collagen types I to III increases and shifts to that of normal skin; fibril size increases to that of a healthy dermis over time^83^ The number of blood vessels in the granulation tissue regresses to the density of unwounded skin^86,110^	Collagen is laid down in a basket-weave pattern similar to that of uninjured skin^114,118,119^ Lower ratio of collagen type I to III^105,106,116,117^ Blood vessel density is also reduced to levels similar to the surrounding tissue^101^

^a^ Most of the research into regenerative healing has been done in nonhuman fetuses, which have different gestational lengths in comparison to humans; thus, the chronology of fetal wound healing in humans is not well established. The animal models used for the time points given are provided.

α-SMA, alpha-smooth muscle actin; ECM, extracellular matrix; FGF, fibroblast growth factor; HGF, hepatocyte growth factor; IL, interleukin; MMP, matrix metalloproteinase; PlGF, placental growth factor; TGF, transforming growth factor; TNF, tumor necrosis factor; VEGF, vascular endothelial growth factor.

It is important to note that many studies on cutaneous wound healing, including those on burns, use animal models.^31,32^ Although animal models may be preferred for reproducibility, control of factors that affect wound healing, costs, and ethical considerations, such models are not completely representative of human wound healing.^31,32^ An additional factor making regenerative healing difficult to study and characterize in humans is that it happens primarily *in utero*.^33^ While this review focuses on human cutaneous wound healing, much of the primary literature cited uses animal models in an attempt to better characterize the process.

### Hemostasis phase

Burn wounds result in significant damage to the surrounding vasculature, which extends out from the initial injury zone and into the zone of stasis, leading to low oxygenation and vessel leakage.^34^ Reparative healing begins with the hemostasis phase. Immediately following a cutaneous injury, a blood clot comprising platelets, cross-linked fibrin, and fibronectin starts to form.^25,35^ Initial clotting prevents excessive blood loss and helps protect the wound from infection.^36,37^ The clot also serves as a temporary ECM that stores growth factors and facilitates the movement of vascular cells, leukocytes, and fibroblasts during the inflammatory stage.^35,38^

In early-stage embryos, hemostasis begins with the formation of a fibronectin clot.^39^ Fibrin is not present in the clot, and platelets have not yet differentiated.^40,41^ Reepithelialization also starts immediately and is completed rapidly.^33,39,40^

### Inflammatory phase

In postnatal healing, the inflammatory phase is initiated by the innate immune response; this phase can last several days and, in cases of severe burns, lead to a hypermetabolic state.^28,42,43^

The response of toll-like receptors to damage-associated molecular patterns released by injured cells triggers the innate immune response and leads to the production of signaling molecules, including tumor necrosis factor alpha (TNF-α), and interleukin (IL)-1, IL-6, and IL-8, which promote the migration of immune cells to the wound.^43–45^ Leukocytes infiltrate the injured tissue by extravasation and help protect the wound from infection.^46^ Neutrophils are involved in phagocytosis and protect the wound from infection by secreting proteases.^46–48^ Neutrophils also secrete cytokines with immunomodulatory functions and chemokines, which signal for additional inflammatory cells to clear debris from the wound.^47,49–51^

The infiltration of immune cells is helped by mast cells, which release histamine and heparin, reducing blood coagulation and increasing fluid accumulation.^45,52,53^ Later in the inflammatory phase, angiogenic growth factors secreted by neutrophils help promote the formation of blood vessels.^49,54,55^ Natural killer cells and plasmacytoid dendritic cells are involved in antimicrobial activity during the innate response, and contribute to angiogenesis and tissue repair during the adaptive response.^56–59^ For more on the role of the immune response in wound healing, see the recent reviews by Cañedo-Dorantes and Cañedo-Ayala and Ellis *et al*.^45,60^

Compared with reparative healing, the inflammatory response in regenerative healing is attenuated.^61,62^ Many of the cells involved in both innate and acquired immunity (e.g., mast cells, macrophages, and neutrophils) are not yet differentiated or are not responsive to the wound.^40,41,62–64^ Therefore, levels of inflammatory cytokines and chemokines are reduced or absent in regenerative healing.^65–67^ In addition, increased expression of anti-inflammatory cytokine IL-10 in postnatal regenerative healing helps decrease the inflammatory response.^68,69^ Although the role of inflammation in regenerative wound healing is still not well understood, inflammation is associated with a fibrotic response, and reduced inflammation is thought to be more conducive to reduced fibrosis and less scarring.^64,69,70^

### Proliferative phase

During the proliferative phase in reparative healing, resident cells in the tissue migrate and proliferate to replace damaged tissue and close the wound.

Macrophages secrete cytokines, chemokines, and growth factors (including vascular endothelial growth factors [VEGFs] and transforming growth factor [TGF]-β) to attract fibroblasts and keratinocytes to the wound.^49,71^ Dermal fibroblasts proliferate and produce an ECM of collagen types I and III, fibronectin, hyaluronic acid, and proteoglycans.^72–76^ Signals from macrophages and other immune cells cause the migration of endothelial cells and endothelial progenitor cells to the wound, where they form new blood vessels.^77,78^ These vessels vascularize the ECM formed by fibroblasts, and together form a highly vascularized stroma of granulation tissue.^74^ Stem cells or mesenchymal progenitor cells from hair follicles, injured nerves, and the bone marrow, and dedifferentiated cells from underlying fat, also contribute to the generation of new tissue.^79–82^

Induced by TGF-β1, fibroblasts in the granulation tissue differentiate into myofibroblasts.^83,84^ The myofibroblasts are rich in alpha-smooth muscle actin (α-SMA) stress fibers, and contract to decrease the wound area.^85^ The contraction of the wound contributes to the vascularization of the granulation tissue by pulling in pre-existing vascular tissue, which increases the size and length of vessels already present.^74,86^ As the wound contracts, keratinocytes from the surrounding tissue and stem cells from the interfollicular epidermis and hair follicles migrate across the wound bed between the granulation tissue and the fibrin clot, epithelializing the wound.^81,87–91^

In fetal regenerative healing, the proliferative phase is initiated quickly after wounding, potentially due, in part, to the early appearance of tenascin, which initiates cell migration and reepithelialization.^33,39,62,92^ Granulation tissue does not form.^93,94^ Instead, fetal wounds show higher levels of proliferating fibroblasts and keratinocytes than postnatal wounds, which produce an organized ECM of fibronectin, tenascin, chondroitin sulfate, and hyaluronic acid, which is similar to the surrounding tissue.^33,39,62,95^ High levels of IL-10 upregulate hyaluronic acid by increasing protein synthesis and decreasing degradation, while also increasing invasion of fibroblasts, which raise the hyaluronic acid content of the ECM.^39,95–98^

Endothelial progenitor cells originate from the bone marrow and contribute to angiogenesis and increased blood circulation.^99^ Both fetal and oral wounds have lower levels of VEGF and associated angiogenesis than in reparative healing; this is possibly because the vessels are more organized and efficient, so fewer are required.^62,100–102^

In fetal wound healing, the levels of antifibrotic TGF-β3 are higher relative to those of profibrotic TGF-β1 and TGF-β2, and fibroblasts do not differentiate into myofibroblasts.^30,41,103–106^ Instead, the fetal fibroblasts contract the ECM to decrease the wound area, but with lower contractile force than postnatal myofibroblasts; this is thought to contribute to the scarless phenotype.^104,107,108^ Reepithelialization happens simultaneously through an actin cable running through the basal cells around the wound edge.^107,109^ This cable contracts and brings the edges of the wound closer together until they seamlessly close the wound.^107,109^ Unlike in postnatal healing, the epidermis moves over the damaged tissue in fetal healing.^33^

### Remodeling phase

In the remodeling phase of reparative wound healing, granulation tissue is replaced with a more organized ECM, resulting in scar tissue that has mechanical properties similar (although not identical) to tissue in the preinjury state.^60^ The density of blood vessels in the granulation tissue regresses to that of unwounded skin.^86,110^

Matrix metalloproteinases secreted by fibroblasts, keratinocytes, and inflammatory cells break down the collagen in the granulation tissue and fibroblasts remodel it into a more permanent structure.^111–113^ The types and arrangement of collagens expressed impact the reparative wound healing process.

In wounds that heal without incident, the ratio of fibrillar collagen type I to collagen type III increases and shifts to that of normal skin, and fibril size increases to that of a healthy dermis over time.^83,114^ Scar tissue is made up primarily of collagen type I arranged in parallel bundles, which is weaker and less pliable than tissue in healthy skin.^30,114,115^

In regenerative healing, IL-10 downregulates the expression of collagen type I through the TGF-β signaling pathway, leading to a lower ratio of collagen type I to collagen type III deposition than in reparative healing.^105,106,116,117^ Blood vessel density is reduced to levels similar to the surrounding tissue.^101^ Furthermore, collagen is deposited in a basket-weave pattern similar to that of uninjured skin, which reduces or eliminates scarring.^114,118,119^ Understanding the key molecular differences between reparative and regenerative wound healing contributes to the identification of factors and therapeutic approaches that may tip the balance toward regenerative wound healing.

## Contraction and Scarring

All reparative healing results in scarring, and large or severe burn wounds can lead to scar contracture and pathological scarring.^120^ The contraction of granulation tissue by myofibroblasts during the proliferative phase is a natural part of the reparative wound healing process.^85^ However, persistence of the myofibroblasts can lead to scar contracture, resulting in pain, physical limitations, and adverse cosmetic results.^120–123^

Pathological scars (i.e., hypertrophic scars [HTSs] and keloids) are also a result of dysregulated healing. HTSs result from an accumulation of fibroblasts and increased collagen production due to reduced apoptosis and collagenase activity and have increased angiogenesis.^49,124–127^ The resulting scar is a mass of cross-linked collagen aligned with the epidermal surface.^49,126,127^ HTSs have a higher ratio of collagen type I to collagen type III than in normal skin, but have more collagen type III than non-HTSs.^49,114,128,129^ There is a high chance of HTS formation if wound healing is delayed by more than 3 weeks.^28^

Unlike HTSs, which typically stay within the wound edges, keloids exhibit uncontrolled growth beyond the borders of the initial wound.^130^ They have multiple layers with varying ratios and levels of organization of collagen types I and III and are difficult to treat.^130,131^ Burn progression, extensive inflammation, and increased mechanical forces are all characteristics of severe burn wounds that contribute to scarring and contracture.

### Burn wound progression and inflammation

Although the inflammatory phase is an essential part of the wound healing process, excessive inflammation during the early stages of wound healing and prolonged inflammation can lead to scarring, fibrosis, and delayed healing.^25,49^ Second-degree (partial thickness) burn wounds can progress to third-degree (full thickness) burn wounds in a matter of days, and this progression is associated with increased inflammation and cell death.^132–135^ In addition, burn wounds with greater total body surface area (TBSA) have greater and more prolonged inflammation than those with smaller TBSA.^42^ The inflammation associated with severe burn wounds leads to a hypermetabolic response.^42,136^

Increased inflammation and the hypermetabolic response delay wound healing, including reepithelialization; delayed reepithelialization can increase scarring and lead to HTSs.^137–141^ There is evidence that preventing burn wound progression and reducing inflammation and the hypermetabolic response can lead to accelerated reepithelialization and wound closure in burn wounds, reducing scarring.^132,138,142,143^ Studies using fetal lamb and human fetal *ex vivo* models have shown that fetal thermal wounds are able to heal without scars or an inflammatory response, although this may be dependent on the size and extent of the burn.^33,63,144^

### Mechanical forces

Although the focus of this review is on the biological mechanisms of wound healing, mechanical forces also play a role and need to be briefly discussed. While the role of mechanical load in wound repair is still not well characterized, it has been associated with an increase in the inflammatory response, the conversion of fibroblasts to myofibroblasts, the specific orientation of myofibroblasts and associated collagen bundles, and fibrosis.^120,125,145–148^ Greater mechanical force is associated with the formation of HTSs and keloids, and repeated mechanical tension can lead to scar contracture.^124,125,149–151^ In addition, the tension caused by contracture may lead to pathological scarring.^146^ Pathologic scarring and contracture are common in burn wounds.^120,152^

Treatment for large burn wounds may involve physical therapy to prevent muscle contraction and stretching or splinting of scars to prevent contracture; this increases mechanical forces and may facilitate the conversion of fibroblasts into myofibroblasts, which can lead to further scarring.^120,153^ Although it is difficult to study the role of mechanical forces in fetal skin, fetal mouse skin has a much lower resting tension than adult skin.^124^ Adult mouse skin has lower resting tension than human skin, and when the tension of human skin is applied to mouse skin, pathologic scarring occurs.^124^ Because fetal skin has a low resting tension, a severely reduced inflammatory response, and no myofibroblasts, it is thought that reduced mechanical tension contributes to scarless healing.^150,153^

Wound healing studies have shown that a reduction of mechanical forces reduces scarring, although therapies that can reduce mechanical forces during burn wound healing are still in development.^145,154^ For an in-depth discussion of mechanical forces and their potential applications in the goal of wound healing, see the recent reviews by Yannas and Tzeranis and Barnes *et al*.^148,153^

## Therapeutics Aimed at Promoting Regenerative Healing in Burn Wounds

The aspirational objective of burn wound treatment is regenerative healing.^17,28,29^ One of the key differences between regenerative and reparative healing is the reduced inflammatory stage in fetal wound healing, secondary to decreased angiogenesis and expression of proinflammatory cytokines and the increased expression of the anti-inflammatory cytokine IL-10.^30,40,62,68,105,155^ Optimal therapeutics for cutaneous wound healing aim to combine cells and cell signaling molecules that modulate inflammation with matrices that allow these cells to respond to endogenous signals in a spatiotemporal manner.^29,156^

Although regenerative healing may never be achieved for burn wounds, basic research continues to provide novel approaches and products aimed at restoring normal skin architecture and reducing adverse outcomes, including infection, delayed reepithelialization, and scarring.^29,157^ The following section will discuss skin substitutes for burn wound healing, give examples of products currently available in the United States, and describe the components being researched for new therapeutic development.

### Skin substitutes for burns

Although autografts are preferred for the treatment of acute burn wounds, they create another wound that can lead to pain, scarring, and reduced quality of life.^158,159^ Additionally, in the case of extensive burn wounds, an individual may not have enough uninjured skin to provide sufficient coverage, creating a need for skin substitutes.^159^ There are many different ways to classify skin substitutes. The categories in Table 2 depend on the origin of the components (human, xenogeneic, and synthetic), the types of components (cells and ECM/scaffold), whether the product contains human cells or tissue, and whether the product is autologous or allogeneic. Depending on the product composition, some products are intended to provide temporary coverage, while others can be used in place of autografts or allografts.

**Table 2.** Categories of Substitute Products or Cellular and/or Tissue-Based Products for Burn Wound Care, with Examples of Currently Used Products

Product category	Description and components	Indications and use^a^	Products (manufacturer)	FDA regulatory pathway^b^	Comments
Synthetic	Wound coverings made up of nonbiological materials. Products may reduce inflammation, increase the rate of epithelialization, and provide a scaffold for tissue regeneration.^217–220^	For temporary coverage of burn wounds.^217,220,221^	BioDegradable Temporizing Matrix/NovoSorb BTM^®^ (PolyNovo Biomaterials Pty Ltd.)^221^	510(k) FRO^221^	For partial-thickness burns.^221^ Contraindicated for clinically infected wounds.^220^
			Suprathel (PolyMedics Innovations, Inc.)^219^	510(k) FRO^217^	For partial-thickness burns.^217^ Contraindicated for clinically infected wounds.^217^
Synthetic and allogeneic nonviable cells ± tissues	Wound coverings made from a synthetic scaffolding embedded with cells or tissue (human or xenogeneic). Products may increase the healing and reepithelialization rate.^222,223^	For temporary coverage of burn wounds or autograft donor sites, depending on the product.^223,224^	Biobrane^™^ (Smith & Nephew)^163^	510(k) FRO^163^	For partial-thickness burns.^223^
			TransCyte^™^ (Organogenesis, Inc.)^c,222^	PMA^224^	For partial- and full-thickness burns.^224^ Contraindicated for patients sensitive to materials with a bovine origin and for clinically infected wounds.^222^
Autologous derived	Skin substitutes made from autologous cells. Supplements or reduces the need for extensive autografting.^225,226^	For the treatment of adult and pediatric burn wounds. These products may be used alone or with autografts.^225,226^	Epicel^®^ (Vericel Corporation)^226^	HDE^226^	For full-thickness burns.^226^ Contraindicated for patients with a history of anaphylaxis to vancomycin, amikacin, and amphotericin; patients sensitive to materials with a bovine or murine origin; and clinically infected wounds.^226^
			ReCell^®^ (Avita Medical, Inc.)^225^	PMA^225^	For partial- or full-thickness burns.^225^ Contraindicated for clinically infected wounds or wounds with necrotic tissue and patients with a hypersensitivity to trypsin or compound sodium lactate solution (Hartmann's Solution).^225^
Placental tissue allografts	Allografts made from dehydrated human amnion and/or chorion membrane. Products may modulate inflammation and support granulation tissue development.^227,228^	For the treatment of burn wounds.^227,228^	AmnioBurn^®^/EpiFix^®^/AmnioFix^®^ (MiMedx Tissue Services, LLC)^163,229^	HCT/P 361^d,163,229^	For partial- and full-thickness burns.^227^ Contraindicated for wounds with an active or latent infection and patients with disorders that would create an unacceptable risk of postoperative complications.^228^
Xenografts	Acellular wound coverings of animal origin, such as bovine or porcine. Products may mitigate the inflammatory response, provide a scaffold for tissue regeneration, and facilitate remodeling.^163,230–234^	For the coverage of burn wounds. Some products may be used in conjunction with standard of care.^230,233,234^	Cytal^®^ Burn Matrix/sheet MatriStem^®^ (ACell, Inc.)^230^	510(k) KGN^163^	For partial-thickness burns.^230^
			Oasis^®^ Burn Matrix (Smith & Nephew)^234^	510(k) KGN^163^	For partial-thickness burns.^234^ Contraindicated for full-thickness burns and patients with sensitivity to porcine material.^234^
			Integra^®^ DRT/ Omnigraft^®^ DRM (Integra Life Sciences Corporation)^233^	PMA^163^	For partial- and full-thickness burns.^233^ Contraindicated for patients with hypersensitivity to bovine collagen or chondroitin materials and clinically infected wounds.^233^
Other human tissue allografts	Acellular human cadaveric dermis allograft.^235^	For the replacement of tissue in burn wound repair.^235,236^	Alloderm^™^ RTM (LifeCell Corporation, an Abbvie company)^237^	HCT/P 361^d,235^	For partial- and full-thickness burns.^d,235,236^ Contraindicated for patients with sensitivity to polysorbate 20 or any antibiotic listed on the package label.^235^
Bioengineered constructs combining cells and/or tissues	Products using bioactive human keratinocytes on a xenogeneic matrix embedded with human fibroblasts. Products may contain stem or progenitor cells.^161,196,238,239^	For the treatment of burn wounds.^196^	Apligraf^®^ (Organogenesis, Inc.)^238^	PMA^238^	For partial- and full-thickness skin ulcers due to venous insufficiency and full-thickness neuropathic diabetic foot ulcers.^e,238^ Contraindicated for clinically infected wounds; patients allergic to bovine collagen; and patients with a hypersensitivity to the Apligraf agarose shipping medium.^238^
			StrataGraft^®^ (Stratatech Corporation, a Mallinckrodt company)^240^	BLA^240^	For partial-thickness burns.^196^ Contraindicated for patients with allergies to murine collagen or products of bovine or porcine origin.^196^

^a^ Products may also be indicated for other wound types.

^b^ Pathways through which currently marketed products for burn wounds have been cleared.

^c^ Formerly Dermagraft-TC.^163^ TransCyte is FDA cleared, but not on the U.S. market.

^d^ 361 Indicates HCT/P products regulated under the Center for Biologics Evaluation and Research under 21 Code of Federal Regulations 1271.3(d)(1) and Section 361 of the Public Health Service Act.^164^ Previously, the FDA exercised enforcement discretion for certain regenerative medicine products so that they did not require premarket review and approval.^166^ As of May 31, 2021, all HCT/P manufacturers were required to file an Investigational New Drug application or a BLA to legally market their products.^165^

^e^ Apligraf is used off-label to treat burns.

BLA, biologics license application; DRM, dermal regeneration matrix; DRT, dermal regeneration template; FDA, U.S. Food and Drug Administration; FRO/KGN, product code for medical devices; HCT/P, human cells, tissues, and cellular and tissue-based product; HDE, Humanitarian Device Exemption; PMA, premarket approval.

Unlike autograft transplantation, skin substitutes can elicit both innate and adaptive immune responses that can lead to rejection of the substitute.^159^ Skin substitutes with bioactive keratinocytes and fibroblasts generate growth factors and cytokines that can elicit a host response that aids in the wound healing process.^159–162^ These substitutes may avoid rejection because they do not contain antigen-presenting immune cells.^160,162^ Substitutes that are acellular, or that have synthetic components, are also less likely to be rejected.^159^ Although many of these products can reduce the need for autografting and improve burn wound repair, consideration of the product composition and its potential immunogenicity are important for clinical practice.

### U.S. Food and Drug Administration regulation of skin substitutes

The products in this section and in Table 2 were chosen because they are U.S. Food and Drug Administration (FDA) cleared and marketed in the United States. While the products may all be considered skin substitutes in clinical practice and for insurance billing purposes, the specific indications for use (and subsequent cost) of these products are determined by the FDA. The FDA regulatory category of a product is determined by the product's components and its level of risk to the patient.^163^ The categories, from lowest to highest level of risk, are human cells, tissues, and cellular and tissue-based products (HCT/Ps), humanitarian use device (HUD), 510(k), premarket approval (PMA), and biologics license application (BLA).^163^

The least-rigorous regulatory category is HCT/Ps.^163,164^ This categorization applies to products that are minimally manipulated and intended for homologous use.^163^ Until recently, the FDA exercised enforcement discretion for some HCT/Ps to give manufacturers time to determine if an application for more rigorous regulation was needed.^165^ As of May 31, 2021, products that do not meet all the requirements for HCT/Ps are required to have a BLA or an investigational new drug application to be marketed.^165,166^ Products that are derived from human and/or animal tissue are regulated under an HUD or PMA, and xenogeneic and synthetic products are regulated under the 510(k) pathway.^163^ A BLA is used for products using human cells and tissues that make a specific action claim.^163^

Tissue-engineered products without live cells may be considered medical devices, which are classified from Class I (lowest risk) to Class III (highest risk), with the level of regulation increasing with the class.^163^ Acellular products are then regulated through two pathways: Class I devices are generally exempt from the 510(k) pathway, Class II devices are usually regulated using the 510(k) pathway, and Class III devices usually require a PMA.^163^

For a more in-depth discussion of FDA regulation of skin substitute products for burns, see Belsky and Smiell.^163^ These products may also be regulated differently outside the United States, and there are additional products that are not indicated for use in U.S. settings. Oberweis *et al*. discussed various regulatory frameworks worldwide for tissue-based products, and the alliance for regenerative medicine lists skin substitutes and where they are approved on their website (https://alliancerm.org/available-products).^167,168^

### Components of regenerative healing used for burn wound research

One of the components of regenerative healing that is being explored for wound healing is stem cells. In regenerative healing, stem cells help mediate wound repair through a variety of molecular signals that promote angiogenesis and ECM formation, recruit endogenous progenitor cells, induce cell differentiation, and reduce inflammation and scarring.^169–177^ Preclinical burn studies in murine models have found that treatment with bone marrow-derived mesenchymal stem cells accelerated wound closure, improved mobility, and reduced fibrosis, and may mediate inflammation, myofibroblast differentiation, and collagen deposition.^178–180^

There are few skin substitute products for burns in clinical trials or on the market that are made with stem cells; the use of autologous or allogeneic cells is more common (Table 2 and Supplementary Table S1). In a real-world study, cultured human keratinocyte autografts, used alone or adjunctively with a wide-meshed autograft, improved survival in patients with large (mean TBSA, 67.5%) burn wounds.^181^ Treatment with autologous epidermal cells on a fibrin matrix reduced contraction and helped maintain skin pliability in patients with burn wounds.^182^ In addition, the incorporation of allogeneic progenitor cells with an acellular matrix may, under appropriate conditions, form an epidermal structure that can respond to local signaling.^161,183,184^

Dermal matrices are another approach to harnessing properties of regenerative healing.^185,186^ Made of synthetic materials or decellularized tissue (human or xenogeneic) (Table 2), these products aim to provide a structured scaffold to guide the development of nonfibrotic tissue.^187^ A study of patients with severe burns found that a decellularized dermal scaffold used over joints helped prevent scarring, and resulted in better conservation of joint function.^188^ In addition, a case study of a patient with extensive HTSs from a burn wound demonstrated that scar excision followed by treatment with a decellularized dermal scaffold and a split-thickness skin graft resulted in limited scar formation, supple skin, and increased range of motion.^189^

Signaling molecules are difficult to incorporate into skin substitutes.^156,190^ When applied without a scaffold, exosomes from mesenchymal stem cells have been shown to reduce inflammation in burn wounds in rats, mirroring the low inflammation seen in regenerative healing.^191^ Platelet-rich plasma, which has high levels of growth factors, is also being explored as a burn treatment.^192–194^ Rats with burn wounds treated with platelet-derived biomaterials showed accelerated healing and fewer inflammatory cells than controls.^195^ While some skin substitute products contain bioactive cells that secrete signaling molecules (Table 2), there are no acellular products for burns with published data showing that they provide signaling molecules at optimal dosages in a relevant spatiotemporal manner.^156,162,190,196^ This may be one aspect for future research to address.

Shaping the future of regenerative medicine for burn wound healing requires further understanding of the processes that have led to the currently available products. The principles and components of regenerative wound healing have not changed over time, but how researchers understand and apply them to the development of new therapeutics has continued to shift. For example, inflammation and a strong immune response are linked to reparative healing, but recent research has linked both processes as also being important to regeneration in animal models.^25,43,49,197,198^ Continued improvement in the understanding of pathways, cells, and signals involved in regenerative healing will allow for the identification of new targets that can be used to drive the development of new therapeutics.^199^

## Summary

Scarring is a natural consequence of reparative healing, including for wounds that heal quickly with minimal interference. All scarring—even in the absence of pathological scarring—results in tissue that does not have the same appearance, strength, or function as the surrounding skin and contributes to physical and psychological burdens for patients with severe burn wounds.^29,42,111^ To ensure long-term patient well-being and quality of life, burn care approaches necessitate advancements that move toward regenerative healing, reduced scarring, and restored strength and function.^29,42^ An increased understanding of the underlying mechanisms of regenerative and reparative healing will contribute to the development of innovative strategies that better incorporate aspects of regenerative healing and improve outcomes for patients with severe burns.^17,29^

## Supplementary Material

Supplemental data
