# Supplementary material for: Healing Mechanisms in Cutaneous Wounds: Tipping the Balance
Source: Tissue Eng Part B Rev. 2022 Oct 11;28(5):1151–67. doi: 10.1089/ten.teb.2021.0114 (PMC9587785; doi:10.1089/ten.teb.2021.0114)
Supplement: Supplemental data [file Supp_TableS1.docx]

SUPPLEMENTARY TABLE 1. SELECTED CURRENT US CLINICAL TRIALS EVALUATING THERAPIES AIMED AT IMPROVING REGENERATIVE WOUND HEALING (CURRENT AS OF SEPTEMBER 15, 2021)

| *Clinical trial #/phase* | *Trial title* | *Intervention* | *Estimated enrollment (status)* | *Estimated completion* |
| --- | --- | --- | --- | --- |
| NCT03695939/phase 1^241^ | Evaluation of safety of Xeno-Skin™ for treatment of severe and extensive, partial- and full-thickness burns | Xeno-Skin™ (alpha-gal-knockout porcine live cells and tissue) ^242^ | 6 (recruiting) | December 31, 2021 |
| NCT04890574/ phase 1^243^ | CellMist™ Autologous Cells to Treat Deep Second-Degree Burns (CELLMIST1) | CellMist™ System (device to deliver autologous skin cells) | 14 (recruiting) | August 26, 2023 |
| NCT04765202/ phase 1/2a^244^ | StrataGraft^®^ overlay of meshed autograft in full-thickness thermal burns (StrataSOMA) | StrataGraft skin tissue overlay of meshed autograft (TE allogeneic cells and matrix)^196^ | 40 (recruiting) | June 2023 |
| NCT03723590/NA^245^ | A clinical evaluation of an esterified hyaluronic acid matrix in burn patients for STSG | Esterified hyaluronic acid matrix | 20 (recruiting) | May 2022 |
| NCT03626701/NA^246^ | RES® prepared with RECELL® compared to standard-of-care dressings of partial-thickness burns in ages 1-16 years | RECELL® autologous cell harvesting device | 19^a^ (Active, not recruiting) | December 1, 2023 |

^a^Actual enrollment.

NA, not applicable; STSG, split-thickness skin graft; TE, tissue engineered.
